# Antimicrobial resistance situation in animal health of Bangladesh

**DOI:** 10.14202/vetworld.2020.2713-2727

**Published:** 2020-12-19

**Authors:** Md. Al Amin, M. Nazmul Hoque, Amam Zonaed Siddiki, Sukumar Saha, Md. Mostofa Kamal

**Affiliations:** 1 Quality Control Laboratory, Department of Livestock Services, Savar, Dhaka-1341, Bangladesh; 2 Department of Gynecology, Obstetrics and Reproductive Health, Faculty of Veterinary Medicine and Animal Science, Bangabandhu Sheikh Mujibur Rahman Agricultural University, Gazipur-1706, Bangladesh; 3 Department of Pathology and Parasitology, Chattogram Veterinary and Animal Sciences University, Chattogram, Bangladesh; 4 Department of Microbiology and Hygiene, Bangladesh Agricultural University, Mymensingh-2202, Bangladesh

**Keywords:** animal health, antibiotic-resistant, antimicrobial resistance, bacteria, veterinary

## Abstract

Antimicrobial resistance (AMR) is a crucial multifactorial and complex global problem and Bangladesh poses a regional and global threat with a high degree of antibiotic resistance. Although the routine application of antimicrobials in the livestock industry has largely contributed to the health and productivity, it correspondingly plays a significant role in the evolution of different pathogenic bacterial strains having multidrug resistance (MDR) properties. Bangladesh is implementing the National Action Plan (NAP) for containing AMR in human, animal, and environment sectors through “One Health” approach where the Department of Livestock Services (DLS) is the mandated body to implement NAP strategies in the animal health sector of the country. This review presents a “snapshot” of the predisposing factors, and current situations of AMR along with the weakness and strength of DLS to contain the problem in animal farming practices in Bangladesh. In the present review, resistance monitoring data and risk assessment identified several direct and/or indirect predisposing factors to be potentially associated with AMR development in the animal health sector of Bangladesh. The predisposing factors are inadequate veterinary healthcare, monitoring and regulatory services, intervention of excessive informal animal health service providers, and farmers’ knowledge gap on drugs, and AMR which have resulted in the misuse and overuse of antibiotics, ultimate in the evolution of antibiotic-resistant bacteria and genes in all types of animal farming settings of Bangladesh. MDR bacteria with extreme resistance against antibiotics recommended to use in both animals and humans have been reported and been being a potential public health hazard in Bangladesh. Execution of extensive AMR surveillance in veterinary practices and awareness-building programs for stakeholders along with the strengthening of the capacity of DLS are recommended for effective containment of AMR emergence and dissemination in the animal health sector of Bangladesh.

## Introduction

Microorganisms are among the man’s best friends and also worst foes. Antimicrobial agents have been extremely important cornerstones of modern medicine in food animal production since the last half of the previous century. Antimicrobials have contributed considerably to the prevention and treatment of infectious diseases in livestock, and some of them have played a very important role in the promotion of animal growth and feed efficiency [1,2]. Antimicrobial resistance (AMR) evolves as a natural consequence of antimicrobial usage (AMU) in multiple sectors such as human health, animal health and animal production, aquaculture, and agriculture [3]. It is considered as a crucial multifactorial and complex global problem because of the rapid emergence and spread of resistant bacteria and associated antibiotic-resistant genes (ARGs) among humans, animals, and the environment [4]. AMR leads to increased morbidity, mortality, disease burden, healthcare expenditure, and reduced livelihoods. It is estimated that an uncontained AMR problem will cause 300 million human deaths globally along with 100 trillion US$ financial losses and 11% fall in livestock productions by 2050 [5,6]. The direct negative impact of AMR in the animal sector is the production losses which ultimately result in reduced food security. Developing countries are more vulnerable to AMR due to inappropriate and overuse of antibiotics, poor quality drugs, non-human use of antibiotics, inadequate drug monitoring and surveillance system, lack of awareness of AMR, and poverty [7].

Terrestrial and aquatic food-animal production industries are expanding rapidly to meet the increasing demand for animal-source nutrition worldwide. This expansion is comparatively greater in low-and middle-income countries (LMICs) than in high-income countries. Rising demand for animal-source nutrition in LMICs is working as driving force for shifting of the small-scale animal farming systems to intensive, large-scale, and specialized commercialization. Non-therapeutic use of antimicrobials for prevention and control of disease or as antimicrobial growth promoters (AGP) are common practices in intensive farm-animal production systems [8]. Use of AGPs at subtherapeutic doses in farm animals exerts selective pressure on circulating beneficial or commensal bacteria which ultimately accelerates the development of AMR [9]. Spill-over of the antibiotic residues, antibiotic-resistant bacteria, and ARGs from the animal farming systems to the surrounding environment and humans are creating a potential public health hazard worldwide [10]. In addition, the expansion of resistant clones, resistome (total resistance to antibiotics and heavy metals), and antibiotic-resistant (ABR) determinants among the animal, human, and environment-associated microbiomes have the potential to alter bacterial population genetics, thereby modifying the structure, and eventually the productivity of microbiomes where antibiotic-resistant bacteria can expand [11].

Bangladesh is a developing country in the Southeast Asian region. The livestock sector of Bangladesh is endowed with 403 million terrestrial animals, which shares about 1.47% of the gross domestic product to the national economy. This sector is also providing full-time and part-time employment to 20% and 50% people of the country, respectively [12]. Both poultry and food-animal farming systems in Bangladesh are diversified from household small farms to medium and large-scale commercial farms [13,14]. Due to the absence of adequate government animal healthcare system, farm owners mostly depend on informal and unqualified healthcare providers for the treatment of their animals. Therefore, irrationally prescribed and easy access to antibiotics leads to misuse, abuse, suboptimal, or overuse of these drugs in farms [13]. Moreover, antibiotics are also used as prophylactic and sometimes as growth promoters, specifically in large-scale commercial farms of Bangladesh [9]. The irrational, suboptimal, or overuse of antibiotics has resulted in the evolution of different species of pathogenic and zoonotic ABR bacteria in animal farming settings of Bangladesh [15-18]. Unhygienic animal husbandry practices in Bangladesh are creating an important risk factor for disseminating these pathogenic and zoonotic ABR bacteria into humans and the environment [17,19].

The World Health Organization (WHO) endorsed a Global Action Plan (GAP) in 2015 based on “One Health” approach for combatting the emerging global threat from AMR [20]. The Food and Agriculture Organization of the United Nations (FAO) and the World Organization for Animal Health (OIE) also developed complementary plans and strategies for the same purpose [9]. In alignment with the WHO GAP guidelines, Bangladesh formulated and approved a National Action Plan (NAP) 2017-2022 for containment of AMR in human, animal, and environment sectors. For successful implementation of NAP, concerted and coordinated actions across the sectors are necessary. For this, detailed information on the current AMR situation in the human, animal, and environment sectors of Bangladesh is a prerequisite [20]. The Department of Livestock Services (DLS), Government of Bangladesh is the mandated body to formulate and execute a surveillance program on AMR and implement NAP strategies in the animal health sector of the country. Although the patterns and extent of AMR development in the animal-borne pathogens of Bangladesh have been reported in some studies [8,18,19,21,22], DLS has not yet looked deep into the matter. Before launching an AMR surveillance program in animal health sector of Bangladesh, it is utmost necessary to understand the knowledge gaps by analyzing nature and extent of the problem. The role of concerned regulating authorities (DLS), and registered veterinary practitioners for implementation of policies for antibiotic stewardship to seize the rising AMR threats is also a fundamental need. In light of emerging aspects of AMR, the present review is, therefore, aimed to fill up the knowledge gaps by reviewing previous studies on the AMR situation in the animal health sector of Bangladesh.

## Literature review

To prepare this review, we conducted a literature review on the AMR situation in the animal health sector of Bangladesh and across the globe from Google Scholar, PubMed, ResearchGate, and Crossref databases. First, we focused the introductory section on the background of AMR situation worldwide, its impacts on livestock and public health sectors, the possible source of AMR in livestock, and current trends in antibiotic usage in Bangladesh. The databases were searched using the term “AMR situation in Bangladesh” with alternative terms “AMR pathogens in animal sector of Bangladesh;” “AMR bacteria in animal sector of Bangladesh;” “AMR bacteria in poultry sector of Bangladesh;” and “AMR bacteria in farm animals of Bangladesh.” “Antibiotic-resistant” or “antimicrobial-resistant” terms were also used as alternative to “AMR.” Searches were filtered for research or review articles published in the English language from January 2011 to June 2020. Grey materials/unpublished documents were searched using *Google* and retrieved from the relevant institutional websites. Studies conducted on the AMR situation in humans, agriculture, aquaculture, or the environment were excluded considering irrelevant to the present study. The articles directly assessed husbandry and medication practices along with antibiotic-resistant bacteria or genes in food-producing or non food-producing animals of Bangladesh were included for general review. The literatures described “in place” institutional and policy structures of DLS to combat AMR burden in Bangladesh were also considered.

The key findings (first authors, year of publication, animal farm type with geographical location, pathogen type, spectrum of resistance with classes of antibiotics, and resistant genes) related to this study were retrieved from the articles. Extracted data were analyzed at 95% confidence level using IBM SPSS Statistics 20 software. To find out the associations and level of significance among different variables, we used Pearson Chi-square test.

## Results

We identified a total of 44 peer-reviewed articles that contained information relevant to the present study. Of these, five articles (Table-S1) reported medication patterns in veterinary practices, 23 articles (Table-S2) reported ABR bacteria and genes prevalent in poultry sector, and 13 articles (Table-S3) reported those prevalent in dairy and other animal sector and three articles (Table-S4) described “in place” institutional and policy structures of DLS to combat ABR burden in Bangladesh. The findings from the review of the selected articles are described below according to the key themes of the study.

**Table-S1:** Articles (n=5) reported the medication patterns in veterinary practices of Bangladesh.

Author, Year	Title of the article	Type of study	Key findings
Roess *et al*., 2015 [13]	Household Animal and Human	Original article	• Rural people share houses and water bodies with animal
	Medicine Use and Animal		• Rural peoples seek animal healthcare from unlicensed village doctors rather than formal-sector healthcare providers
	Husbandry Practices in Rural		
	Bangladesh: Risk Factors for Emerging Zoonotic disease and antibiotic resistance		•Animal medicines, including antimicrobial drugs are being used extensively in rural households of Bangladesh
Saiful Islam *et al*.,2016 [24]	Antibiotic Usage Patterns in Selected Broiler Farms of Bangladesh and their Public Health Implications	Research article	•43.8% farmers used antibiotics for therapeutic purpose, 31.5% for prophylaxis and 8.2% for growth promotion • >60% farmers used antibiotics without any prescription
			• Residual antibiotics were detected in 26% of broiler meat
			•Fluoroquinolones (68.4%) were the most commonly detected antibiotics
Khan *et al*., 2018 [26]	Study on indiscriminate use of antibiotics in poultry feed and residues in broilers of Mymensingh city in Bangladesh	Research article	• 17.5% of the broiler sellers used antibiotics to prevent unwanted mortality • 32.5% of the sellers depended on feed sellers and 20% on registered veterinarian for antibiotic use
			• The highest percentages of antibiotics used in poultry feed was enrofloxacin (46.67%), followed by ciprofloxacin (30.00%) and amoxicillin (23.33%)
			• 100% broiler liver and 20% breast meat contained antibiotic residues
Ferdous *et al*., 2019 [25]	Assessing farmers’ perspective on antibiotic usage and management practices in small-scale layer farms of Mymensingh district, Bangladesh	Research article	• 94.16% of farmers used antibiotics without maintaining the withdrawal period • Farmers used ten different types of antibiotics of seven classes
			•Farmers used antibiotics of Watch group (49%), Reserve group (8%) and Las Resort group (73%)
Masud *et al*., 2020 [23]	Drivers of Antibiotic Use in Poultry Production in Bangladesh: Dependencies and Dynamics of a Patron-Client Relationship	Original research	• Poultry dealers provide credit and information for small-scale poultry farmers to initiate and operate their business •Farmers are obliged to buy poultry feed and medicine from their dealer
			•All farms apply multiple antibiotics to poultry throughout the production cycle, including banned antibiotics like colistin sulfate
			•Dealers are the main influencers on antibiotic use in farms

**Table-S2:** Articles (n=23) reported antimicrobial-resistant pathogens and genes in poultry sector of Bangladesh.

Author, Year	Title of the article	Type of study	Key findings
Mahmud *et al*., 2011 [38]	Prevalence of *Salmonella* Serovars and AMR Profiles in Poultry of Savar Area, Bangladesh	Research article	•The prevalence of *Salmonella* was recorded in 21.1% poultry farms
			•*Salmonella* isolates showed resistance against 5-10 antibiotics
Hasan *et al*.,2011 [28]	High Prevalence of antibiotic resistance in Pathogenic *E. coli* from Large- and Small-Scale Poultry Farms in Bangladesh	Research article	• More than 55% *E. coli* isolates were resistant to at least one or more antibiotics • 36.6% of the isolates showed MDR
Hasan *et al*.,2012 [31]	Antimicrobial Drug-Resistant *E. coli* in Wild Birds and Free-range Poultry, Bangladesh	Research article	• 22.7% *E. coli* isolates were MDR
Jakaria *et al*., 2012 [29]	Prevalence, Characteristics and antibiogram profiles of *E. coli* Isolated from Apparently Healthy Chickens in Mymensingh, Bangladesh	Research article	•*E. coli* isolates were sensitive to ciprofloxacin, gentamicin and cephalexin • Resistant to streptomycin, tetracycline, amoxicillin, and nalidixic acid
Singh *et al*.,2012 [35]	Isolation and detection of antibiotic sensitivity pattern of *E. coli* from Ducks in Bangladesh and Nepal	Research article	• Isolates were highly sensitive to ciprofloxacin, chloramphenicol and amoxicillin • Moderately sensitive to nalidixic acid, cephalexin, and co-trimoxazole
			• Less sensitive to kanamycin
Hosain *et al*., 2012 [39]	Prevalence and antibiogram profiles of *Salmonella* Isolated from Pigeons in Mymensingh, Bangladesh	Research article	• 80-90% *salmonella* isolates were resistant to amoxicillin, ampicillin, erythromycin, and tetracycline
			• 60-80% *salmonella* isolates were sensitive to ciprofloxacin, sulfamethoxazole, chloramphenicol, kanamycin, gentamicin, and nalidixic acid
Sultana *et al*., 2012 [32]	Multidrug-resistant bacteria in the respiratory tract of apparently healthy quails	Research article	• MDR *E. coli,* *Salmonella* spp., *Pasteurella* spp., *Bacillus* spp., and *Staphylococcus* spp. were identified in quails
Dey *et al*., 2013 [33]	Prevalence of multidrug-resistant *E. coli* in Pigeon in Mymensingh, Bangladesh	Research article	• Pigeons are reservoir of MDR *E. coli* • 70-90% *E. coli* is resistant to sulfamethoxazole, tetracycline, and amoxicillin
Nandi *et al*.,2013 [41]	Prevalence and Characterization of Multidrug-Resistant Zoonotic *Enterobacter* spp. in Poultry of Bangladesh	Original article	• MDR and zoonotic *Enterobacter* spp. are prevalent in poultry farms of Bangladesh
Lutful Kabir *et al*., 2014 [44]	Isolation, Identification and AMR Patterns of *Campylobacter* Species from Broiler Meat Sold at KR Market of Bangladesh Agricultural University Campus, Mymensingh	Research article	• MDR *C. jejuni* and *C. coli* are prevalent in broiler meat
Islam MN *et al*., 2014 [43]	Detection of *S. aureus* in Frozen Chicken Rinse through Bacteriological and Nuc Gene Specific PCR Methods and their drug resistance Patterns in Southern Chittagong, Bangladesh	Research article	• *Staphylococcus* isolates showed 100% resistance to ampicillin, more than 80% were resistant to oxytetracycline, doxycycline, and amoxicillin. Ciprofloxacin showed 77.5%, Cephalexin 38.33%, and Gentamycin showed the least resistance 13.33%
Al-Salauddin *et al*., 2015 [36]	Isolation, identification, and antibiogram studies of *Salmonella* species and *E. coli* from boiler meat in some selected areas of Bangladesh	Research article	• *Salmonella* and *E. coli* isolates from broiler meat were resistant to amoxicillin (80-82%), erythromycin (82-85%), and tetracycline (24-68%)
Saifuddin *et al*., 2016 [8]	Molecular characterization and AMR patterns of *Salmonella* spp. and E. coli of laying chicken	Research article	• 70-100% of *Salmonella* spp. and *E. coli* were resistant to β-lactam antibiotics • 60-90% isolates of both species were susceptible to ciprofloxacin and gentamicin
Parvej *et al*.,2016 [40]	Prevalence and characterization of multidrug-resistant *Salmonella* Enterica serovar Gallinarum biovar Pullorum and Gallinarum from chicken	Research article	• MDR and highly clonal *Salmonella* Enterica are prevalent in commercial layers of Bangladesh
Shahjada *et al*., 2017 [27]	Bacteria causing omphalitis in newly hatched chicks from broiler and layer flocks and their antibiotic profiles	Research article	•*E. coli* (28%), *Salmonella* spp. (38%), *Staphylococcus* spp. (34%) from broiler chicks and *E. coli* (32%), *Salmonella* spp. (36%), *Staphylococcus* spp. (32%) from layer chicks were identified
			• *Staphylococcus* spp. were resistant to ampicillin (100%) and kanamycin (100%); *E. coli* isolates were resistant to ampicillin (80-100%), amoxicillin (60-100%) and kanamycin (80%); *Salmonella* spp. were resistant to ampicillin (100%), kanamycin (80%), and tetracycline (60%)
Momtaz *et al*., 2018 [22]	Occurrence of Pathogenic and Multidrug-Resistant *Salmonella* spp. in Poultry Slaughter-House in Bangladesh	Original article	•MDR and pathogenic *Salmonella* spp. are circulating in poultry slaughterhouses • These MDR Salmonella spp. are potential threat to public health
Sarker *et al*., 2019 [30]	Antibiotic resistance of *E. coli* isolated from broilers sold at LBMs in Chattogram, Bangladesh	Short Communication	• *E. coli* isolates were 100% resistant to ampicillin and tetracycline • All *E. coli* isolates carried blaTEM, tetA, and Sul2 genes
M.S. Hasan *et al*., 2019 [42]	Complete genome arrangement revealed the emergence of a poultry origin superbug *C. portucalensis* strain NR-12	Short Communication	• *C. portucalensis* NR-12 is an emerging superbug from poultry • Complete genome of *C. portucalensis* NR-12 harbored 13 acquired AMR gene markers
			• *C. portucalensis* NR-12 is resistant to 8 different antibiotics from six antimicrobial groups
Neogi *et al*.,2020 [21]	Risk of multidrug-resistant *Campylobacter* spp. and residual antimicrobials at poultry farms and LBMs in Bangladesh	Research article	• 49 and 42% strains of *Campylobacter* spp. were identified as MDR bacteria • Residual antimicrobials (oxytetracycline, ciprofloxacin, and enrofloxacin) were detected in broiler liver and meat
Amin *et al*., 2020 [15]	Occurrence and genetic characteristics of *mcr*-1 positive colistin-resistant *E. coli* from poultry environments in Bangladesh	Original article	• The emerging plasmid-mediated colistin resistance gene *mcr*-1 • All ESBL-producing *E. coli* carrying mcr-1 were resistant to multiple classes of antibiotics
Shanzida *et al*., 2020 [16]	Molecular Detection of Multidrug-resistant *Salmonella* Species Isolated from Broiler Farm in Bangladesh	Original article	• Overall prevalence of *Salmonella* was 35% in broiler farms • *tetA, floR, bla*_TEM-1_*, aadA1, and intl1* genes were detected in the MDR *Salmonella* isolates
Parvin *et al*.,2020 [34]	AMR Pattern of *E. coli* Isolated from Frozen Chicken Meat in Bangladesh	Original article	• MDR and XDR *E. coli* are prevalent in frozen chicken sold at Super shops • ESBL genes blaTEM, blaSHV and *bla*_CTX-M-2_ are prevalent MDR and XDR *E. coli*
Dutta *et al*.,2020 [37]	Acquisition of Plasmid-Mediated Colistin Resistance Gene *mcr*-1 in *E. coli* of Livestock Origin in Bangladesh	Research article	• *E. coli* isolated from humans, animals, environment, and food samples in Bangladesh was MDR (70.9%)
			• *mcr*-1 gene was detected in *E. coli* isolates of poultry source
			•*mcr*-1 sequences revealed that this gene evolved locally

AMR=Antimicrobial resistance, MDR=Multidrug resistance, LBMs=Live bird markets, *E. coli=Escherichia coli*, ESBL=Extended-spectrum β-lactamase, *C. portucalensis=Citrobacter portucalensis, S. aureus=Staphylococcus aureus, C. jejuni=Campylobacter jejuni, C. coli=Campylobacter coli*

**Table-S3:** Articles (n=13) reported antimicrobial-resistant pathogens and genes in dairy and other animals of Bangladesh.

Author, Year	Title of the article	Type of study	Key findings
Rahman *et al*., 2013 [47]	Isolation and Identification of Bacterial Agents Causing Clinical Mastitis in Cattle in Mymensingh and Their Antibiogram Profile	Short communication	•*Staphylococcus* spp. (62.5%), *Streptococcus* spp. (56.25%), *Bacillus* spp. (37.5%), and *E. coli* (31.25%) were identified as causal agents of mastitis
			•Chloramphenicol and erythromycin were found effective for the treatment of mastitis
Ahmed *et al*., 2013 [19]	The Central Cattle Breeding and Dairy Farm, Bangladesh waste contributes in emergence and spread of aminoglycoside-resistant bacteria	Research article	•Aminoglycoside antibiotics (Gentamycin, Kanamycin, and Streptomycin) resistant bacteria are spreading from dairy farm and veterinary clinics to environment
Hossain *et al*., 2013 [52]	Isolation, identification, and antibiogram study of *Pseudomonas aeruginosa* from Cattle in Bangladesh	Original article	•*Pseudomonas aeruginosa* isolates were resistant to ampicillin, oxytetracycline, tetracycline, and amoxicillin
Islam *et al*., 2013 [53]	Prevalence and AMR patterns of *Vibrio Cholerae* from Bangladesh	Research article	• All of the *V. cholerae* isolates milk, water, and feces of dairy farm were found MDR
	Agricultural University dairy farm		•MDR *V. cholerae* isolates were resistant to erythromycin (95.23), azithromycin (76.9%), and ampicillin (52.38%)
Haque *et al*., 2014 [48]	Identification, Molecular Detection and Antibiogram Profile of Bacteria Isolated from California Mastitis Test Positive Milk Samples of Crossbred Cows of Satkhira District in Bangladesh	Research article	•*S. aureus* (49.09%), followed by *E. coli* (27.27%), coagulase-negative *Staphylococcus* (CNS) spp. (18.18%) and *Bacillus* spp. (5.45%) were responsible for mastitis
			•*S. aureus* isolates were found resistant to 5 antibiotics and E. coli to 9 antibiotics
			•*E. coli* isolates were completely resistant to ampicillin (100%) and amoxicillin (100%)
Rana *et al*., 2016 [54]	Antibiotic Resistance, Microbial and Morphological Changes of Marketed Bovine Liver at Different Time Interval from Chittagong, Bangladesh: A Public Health Concern	Research article	•Bovine livers sold at retail meat shops are contaminated with high extent of MDR bacteria
Islam *et al*., 2016 [50]	Isolation and epidemiology of multidrug-resistant *E. coli* from goats in Cox’s Bazar, Bangladesh	Original article	•Overall prevalence of *E. coli* in the rectal swabs of goats was 52%
			•Among the *E. coli* isolates, 39.74% were resistant to 3-8 subclasses of antibiotics
Das Gupta*et al*., 2017 [49]	Prevalence and antibiotic susceptibility pattern of *E. coli* in cattle on Bathan and intensive rearing system	Research article	•The prevalence of *E. coli* was significantly high in cattle under intensive farming than cattle on Bathan
			•Antibiotic-resistant *E. coli* isolates are present in cattle of different management systems
Hoque *et al*., 2018 [46]	Molecular characterization of *S. aureus* strains in bovine mastitis milk in Bangladesh	Research article	•*S. aureus* isolates from bovine subclinical mastitis milk showed highest resistance to oxytetracycline (74.5%), followed by oxacillin (55.9%), ciprofloxacin (49.6%), amoxicillin (42.0%), trimethoprim/sulfamethoxazole (30.0%), and to a less extent to gentamicin (17.9%), penicillin (11.0%), and erythromycin (8.2%)
Sobur *et al*., 2019 [17]	Antibiotic-resistant *E. col*i and *Salmonella* spp. associated with dairy cattle and farm environment having public health significance	Research article	•Dairy farm and their environmental components carry antibiotic-resistant pathogenic *E. coli* and *Salmonella* spp.
Jalal *et al*., 2019 [51]	ABR zoonotic bacteria in Irrawaddy squirrel (*Callosciurus pygerythrus*)	Original article	•Irrawaddy squirrels harbor several types of zoonotic pathogenic AMR bacteria
Siddiki *et al*., 2019 [45]	Comparison of Bacterial Pathogens Associated with Different Types of Bovine Mastitis and their antibiotic resistance Status in Bangladesh	Original article	•*Staphylococcus* spp., *Streptococcus* spp., *Bacillus* spp., and *E. coli* are associated in 78.54%, 80%, and 71.67% mastitis cases as a single and in 21.46%, 20%, and 28.33% as mixed infection, respectively
			•The isolates were resistant to streptomycin (70-100%), amoxicillin (30-100%), and ampicillin (100%)
Hoque *et al*., 2020 [18]	Insights into the Resistome of Bovine Clinical Mastitis Microbiome, a Key Factor in Disease Complication	Original research	•76.2% of six selected pathogens were highly resistant to tetracycline, doxycycline, nalidixic acid, ampicillin, and chloramphenicol
			•Among the RATC functional groups, MDR to efflux pumps (MREP, 28.6%), CmeABC operon (8.9%), resistance to fluoroquinolones (RFL, 6.2%), mdtABCD cluster (5.5%), methicillin resistance in Staphylococci (MRS, 3.8%), BlaR1 regulatory family (BlaR1, 3.4%), MexE-MexFOprN (2.4%), and beta-lactamase resistance (BLAC, 2.2%) were the dominating ARGs found in CM milk microbiomes

AMR=Antimicrobial resistance, MDR=Multidrug resistance, ARGs=Antibiotic resistance genes, *E. coli=Escherichia coli, S. aureus=Staphylococcus aureus*, *ABR=Antibiotic-resistant*

**Table-S4:** Articles (n=3) described institutional and policy structure of the DLS of Bangladesh.

Author, Year	Title of the article	Type of study	Key findings
Zellweger *et al*., 2017 [3]	A current perspective on AMR in Southeast Asia	Review	•Multiple drivers along with loosely regulated access to antimicrobials are responsible for AMR development in the region
Samuel *et al*., 2019 [6]	Veterinary AMR containment in Bangladesh: Evaluating the NAP and scoping the evidence on implementation	Review	•Policy gaps, including an explicit financing modality and specifications for antimicrobial stewardship (AMS) exist in the veterinary sector
			•Rigorous operational, monitoring and evaluation frameworks weakness in veterinary sector
Hoque R *et al*., 2020 [20]	Tackling AMR in Bangladesh: A scoping review of policy and practice in human, animal and environment sectors	Research article	•Bangladesh has developed NAP for containment of AMR
			•Lack of functional mechanism for implementation or coordination, lack of adequate financial, and institutional resources for relevant capacity, and means for infection prevention and control and building awareness and political commitment are the barriers to implement NAP

AMR=Antimicrobial resistance, NAP=National action plan, DLS=Department of Livestock Services

### Medication patterns in veterinary practices of Bangladesh

In rural Bangladesh, 57.7% of households own livestock, including large animals (cattle and buffalo), small ruminants (sheep and goats), and poultry (back yard and commercial). Government veterinary healthcare providers rarely (9.7%) visit these households. In the absence of adequate veterinary healthcare service, animal owners in rural areas avail low-cost animal health care from pharmacies and unlicensed village doctors (82.5%). In addition to feed additives, animal owners use antibiotics, largely at suboptimal doses on suggestions of the informal animal healthcare providers [13]. The prescribing and dispensing of antibiotics in animal sectors of Bangladesh are neither lawfully regulated nor their use lawfully audited [20].

Medication practices in poultry sector of Bangladesh are very complex. In commercial poultry farms, especially in small and medium-scale settings the poultry and feed dealers are the main influencers on antibiotic usage. The poultry and feed dealers provide financial support and farming related technical information to the farmers to initiate and operate their farms. Thereby, the farmers become obliged to buy poultry chicks, feed, and medicine from the dealers. Sales representatives of the pharmaceutical companies are another influencing group in poultry farming systems. In addition to providing product information, sales representatives also provide treatment advice directly to the farmers. Recently, qualified veterinary doctors appointed by the hatcheries, feed companies and pharmaceutical companies are providing poultry management and treatment services to the farmers [23].

The study revealed that the incidence of active infections, high mortality rates, and an aspiration to prevent disease were the major drivers of antimicrobials use in poultry farms. Broiler farmers use antibiotics for therapeutic purposes, for prophylaxis, and also for growth promotion. The majority of the farmers (>60%) use antibiotics without any prescription from veterinary doctors [24]. Layer farmers are also not aware of prudent use of antibiotics. They use antibiotics to prevent egg production fall, for treatment purposes and also for prophylaxis in suboptimal doses. Most of the small-scale layer farmers (94.16%) use antibiotics without prescription and do not maintain the withdrawal period of drugs [25]. Besides the farms, the poultry meat sellers in live bird markets (LBMs) also use different types of antibiotic to prevent unwanted mortality [26].

The small and medium-scale poultry farmers apply multiple antibiotics, even banned antibiotics to poultry throughout the production cycle following the suggestions of the poultry dealers [23]. As many as 19 and ten different types of antibiotic usage have been recorded in the broiler and layer farms, respectively. The most commonly used antibiotics in both types of farming systems are ciprofloxacin, ampicillin, amoxicillin, trimethoprim, oxytetracycline, tylosin tartrate, tiamulin, norfloxacin, enrofloxacin, doxycycline, and colistin sulfate [24,25].

### Antibiotic-resistant bacteria reported in the poultry sector

A total of eight ABR bacterial species were reported in layer, broiler, backyard poultry, ducks, pigeons, and quails in nine different districts of Bangladesh (Table-1) [8,15,16,22,27-44]. ABR *Escherichia coli* was identified in different types of birds from both conventional and unconventional farming systems in different areas of Bangladesh. Newly hatched broiler and layer chicks in hatcheries of Gazipur district experienced navel infection with ABR *E. coli* with a higher degree of resistance against ampicillin (80-100%), amoxicillin (60-100%), and kanamycin (80%) [27]. MDR *E. coli* isolates recovered from dead, sick, and apparently healthy broilers of Gazipur, Mymensingh, and Chattogram districts showed a varying magnitude of resistance against tetracycline (45.5-100%), ampicillin (100%), trimethoprim-sulfamethoxazole (26.7-94.59%), nalidixic acid (25.7-91.89%), ampicillin (25.7%), streptomycin (20.8%), and ciprofloxacin [28-30]. Of these, tetracycline, ampicillin, nalidixic acid, and trimethoprim-sulfamethoxazole were the antibiotics against which *E. coli* from all the broiler sources developed resistance. All *E. coli* isolates from apparently healthy broilers of LBMs in Chattogram city carried *bla*_TEM_, *tet*A, and *Sul*2 multidrug-resistant genes. Likewise, MDR *E. coli* was also reported in layer chickens with resistance against the same type of antibiotics as in broiler from different areas of Bangladesh [8,29]. Ubiquitous dissemination of MDR *E. coli* with resistance against tetracycline (100%), fluoroquinolone (85%), sulfamethoxazole-trimethoprim (85%), aminoglycosides (71%), and nitrofurantoin (21%) was identified in backyard poultry of Bangladesh. These extended-spectrum β-lactamases (ESBL) producing *E. coli* from backyard poultry carried *mcr-1* plasmid and resistant genes *bla*_CTX-M-group-1_, *bla*_TEM_-1 (ESBL), *qnrB* (fluoroquinolone), and *rmtB* (aminoglycoside) [15]. MDR and ESBL-producing *E. coli* was also found prevalent in ducks, geese, quails, and pigeons from unconventional commercial farms and LBMs [31-33]. The *E. coli* isolates from the birds of unconventional farms were resistant against tetracycline (90%), amoxicillin (70%), sulfamethoxazole (90%), and other antibiotics commonly used in broiler and layer farms. Besides the farming settings, alarmingly MDR and XDR *E. coli* with resistance against oxytetracycline (93%), amoxicillin (91.9%), ampicillin (89.5%), trimethoprim–sulfamethoxazole (88.4%), tetracycline (84.9%), and carbapenems (89.6%) were reported in frozen chicken meats from various parts of Bangladesh. ESBL genes *bla*_TEM_, *bla*_SHV_, and *bla*_CTX-M-2_ were prevalent in these MDR and XDR *E. coli* [34].

Multidrug-resistant (MDR) *Salmonella* species were reported from various types of birds reared in different farming systems. Day-old chicks, dead, sick, and even apparently healthy layer birds harbored MDR *Salmonella* with resistance against penicillin-G (90-100%), ampicillin (100%), amoxicillin (98-100%), tetracycline (93%), nitrofurantoin (78%), sulfamethoxazole (60%), gentamycin (46%), and ciprofloxacin (20-40%) [8,36,38,40]. MDR *Salmonella* spp. was also identified in cloacal swabs, litter, and feed samples of broiler farms with resistance against a range of antibiotics and the antibiotic resistance was carried by *tetA* (97.14%), *floR*, *bla*_TEM-1_, and *aadA1* (77.14%) genes [16]. Two studies reported the dissemination of MDR *Salmonella* in unconventional farming systems. Apparently healthy quails and pigeons were found to carry MDR *Salmonella* with resistance against amoxicillin (90%), ampicillin (80%), erythromycin (80%), tetracycline (60%), chloramphenicol (40%), kanamycin (40%), gentamicin (40%), nalidixic acid (40%), sulfamethoxazole (30%), and ciprofloxacin (20%) [32,39]. In LBM, broiler meat, poultry slaughterers’ hand, and poultry residual containers harbored MDR *Salmonella* with the highest resistance to imipenem (83.33%) [22,36]. *Salmonella* from all of the poultry-associated sources has already developed resistance against a broad range of antibiotics, including ampicillin, amoxicillin, tetracycline, and gentamycin (Table-1).

**Table-1:** Antibiotic-resistant bacterial species reported in poultry and other birds of Bangladesh from January 2011 to June 2020.

Name of bacteria	Source	Area (District)	Spectrum of antibiotic resistance (%)	References
*E. coli*	Apparently healthy, sick and dead broiler, layer and domestic birds; ducks, and geese; apparently healthy Japanese quails; broiler meat; newly hatched chicks from broiler and layer flocks	Dhaka, Gazipur, Mymensingh, Chattogram, Rajshahi, Sylhet, Sherpur	Penicillin (100%), tetracycline (45.5-100%), oxytetracycline (93%), trimethoprim-sulfamethoxazole (26.7-94.5%), nalidixic acid (25.7-91.89%), ampicillin (25.7-100%), amoxicillin (70-92%), cephalexin (80%), streptomycin (20.8%), fluoroquinolone (85%), ciprofloxacin (12.9-40%), chloramphenicol (8.9%), nitrofurantoin (2-21%), aminoglycosides (71%), gentamicin (2-40%), kanamycin (80%) and carbapenems (90%) ESBL-producing *E. coli* carried mcr-1 plasmid and resistant genes *bla_CTX-M-1_*, *bla_CTX-M-2_*,*bla_TEM-1_,* *blaSHV*, *qnrB* (fluoroquinolone) and *rmtB* (aminoglycoside)	[8,15,27-37]
*Salmonella* spp.	Apparently healthy, sick and dead layer and broiler chickens; litter and feed samples from broiler farms; apparently healthy pigeons of LBMs, farms and villages; Apparently healthy Japanese quails; Poultry slaughterer’s hand and poultry residual container of poultry-slaughter houses; broiler meat; newly hatched chicks from broiler and layer flocks	Dhaka, Gazipur, Sherpur, Mymensingh and Chattogram	Penicillin-g (90-100%), ampicillin (82.85-100%), amoxicillin (90-98%), cephalexin (70%), streptomycin (77.14%), tetracycline (93-97.14%), chloramphenicol (94.28%), cotrimoxazole (80%), nitrofurantoin (50-78%), sulfamethoxazole (60%), gentamycin (40-46%), erythromycin (80%), nalidixic acid (40-66.6%), kanamycin (40-80%), doxycycline (66.66%), ciprofloxacin (20-40%), and imipenem (83.33%) Resistance was mediated by *tetA* (97.14%), *floR* (94.28%), *bla*_TEM-1_ (82.85%), *aadA1* (77.14%) genes	[8,16,22,27,32,36,38-40]
*Enterobacter* spp.	Layer poultry	Dhaka	Penicillin (100%), rifampicin (100%), ampicillin (94.4%), clindamycin (94.4%), erythromycin (94.4%), vancomycin (88.9%), sulfonamides (72.2%), imipenem (66.6%), streptomycin (55.6%), nitrofurantoin (33.3%), doxycycline (33.3%), tetracyclines (33.3%), and gentamicin (5.6%)	[41]
*C. portucalensis*	Layer poultry	Narayanganj	Resistant to polymyxin, sulfonamide, tetracycline, aminoglycoside, fluoroquinolones, and macrolide antimicrobial groups. Complete genome harbored following 8 AMR genes: *dfrA12* (trimethoprim); *sul1* and *sul2* (sulfonamide); *mph* *(A)* (macrolide); *tet (A)* (tetracycline); *qnrS1* and *qnrB13* (fluoroquinolone); *blaCMY-39* ESBL, *blaTEM-176* (non-ESBL) and *aadA2*, a*ph (30)-Ia*, *aph (300)-Ib, aph (30)-Ic, aph (30)-Id, strA, strB* (aminoglycoside)	[42]
*Pasteurella* spp.; *Bacillus* spp.	Apparently healthy Japanese quails	Mymensingh	Resistant to erythromycin, sulfamethoxazole and tetracycline (Quantitative data not available)	[32]
*Staphylococcus* spp.	Apparently healthy Japanese quails; frozen chicken rinse; newly hatched chicks from broiler and layer flocks	Mymensingh, Gazipur, Chattogram	Resistant to amoxicillin and ampicillin (100%) and kanamycin (100%), oxytetracycline (100%), doxycycline (80%), and ciprofloxacin (77.5%)	[27,32,43]
*Campylobacter* spp.	Hatcheries, broiler farms, and LBMs; broiler meat	Mymensingh, Gazipur, and Tangail	*C. jejuni*: Ampicillin (100%), amoxicillin (58-66%), tetracycline (60-72.72%), streptomycin (21-25%), erythromycin (44-59.09%), azithromycin (19-20%), nalidixic acid (77.27%), ciprofloxacin (41-45.45%), and norfloxacin (25-54.54%) 49-86.36% *C. jejuni* isolates were MDR *C. coli:* Ampicillin (100%), amoxicillin (43-61%), tetracycline (43-66.67%), gentamycin (10-22.22%), erythromycin (43-77.77%), azithromycin (18-19%), nalidixic acid (44.44%), ciprofloxacin (23-26%), and norfloxacin (49-66.67%) 42-100% *C. coli* isolates were MDR	[21,44]

MDR=Multidrug resistance, LBMs=Live bird markets, *E. coli=Escherichia coli*, ESBL=Extended-spectrum β-lactamase, *C. portucalensis=Citrobacter portucalensis, C. jejuni=Campylobacter jejuni, C. coli=Campylobacter coli*

MDR nosocomial opportunistic pathogen *Enterobacter* spp. were documented in layer poultry with resistance against a range of antibiotics used in both animals and humans [41]. Another potential human pathogen *Citrobacter portucalensis* from layer poultry also showed resistance against a wide range of antibiotics mediated by eight AMR genes in its genome [42]. Moreover, *Pasteurella*, *Bacillus*, and *Staphylococcus* species isolated from apparently healthy Japanese quails and newly hatched broiler and layer chicks showed resistance against commonly used antibiotics (Table-1). Foodborne pathogen *Staphylococcus aureus* which showed resistance against a number of antibiotics was identified in the frozen chicken rinse [43].

Reports on the prevalence of antimicrobial-resistant crucial foodborne pathogen *Campylobacter* from different areas of Bangladesh revealed the contamination of broiler meat with MDR *Campylobacter jejuni* and *Campylobacter coli* with varying degree of resistance against several antibiotics (Table-1). In addition, oxytetracycline, ciprofloxacin, and enrofloxacin residues were detected in the majority of broiler liver and meat samples, some of which had a concentration above the acceptable limit [21].

### Antibiotic-resistant bacteria prevalent in dairy and other animals

Different species of ABR bacteria have been documented from dairy, goat, and wild animal sources in different areas of Bangladesh. MDR *E. coli* have been reported in cattle and goat of intensive and free-range farming systems as well as in milk of cows, milkers’ hands, and different components of farm environment (Table-2). Along with resistance against antibiotics commonly used in veterinary practices, *E. coli* isolated from milk, milkers’ hands, and farm environment carried *tetA* and *SHV* resistance genes and developed extreme resistance against azithromycin (100%) which is recommended to use in humans only [17]. Milk of mastitis-affected cows was identified as another important source of MDR *E. coli* with resistance against ampicillin (100%), amoxicillin (30-100%), and streptomycin (70-100%) [18,45]. Besides *E. coli*, milk of mastitis-affected cows was also a source of antibiotic-resistant *Bacillus*, *Streptococcus*, *Staphylococcus*, *Klebsiella*, *Enterobacter*, and *Shigella* spp. which developed resistance against several antibiotics (Table-2) [17-19,45-54]. In a study of bovine subclinical mastitis milk, *S. aureus* isolates showed the highest resistance to oxytetracycline (74.5%), followed by oxacillin (55.9%), ciprofloxacin (49.6%), amoxicillin (42.0%), trimethoprim/sulfamethoxazole (30.0%), and to a less extent to gentamicin (17.9%), penicillin (11.0%), and erythromycin (8.2%) [46]. The disposal of farm waste directly into the environment contributes in the ABR bacteria pollution and ultimately poses a health hazard to both farm animals and humans [19].

**Table-2:** Antibiotic-resistant bacterial species reported in dairy and other animals of Bangladesh from January 2011 to June 2020.

Name of bacteria	Source	Area (District)	Spectrum of antibiotic resistance (%)	Reference
*E. coli*	Cattle of intensive and free-range farming systems; cow dung, milk, milkers’ hand wash, soil, water, and vegetables of dairy farms; milk from mastitis affected cows; feces of goats; wild Irrawaddy squirrels	Dhaka, Mymensingh, Chattogram, Sirajganj, Satkhira, Rajshahi, Cox’s bazar and Bandarban	Azithromycin (100%), amoxicillin (60.26-100%), ampicillin (65.38-100%), cephalexin (53.8%), nalidixic acid (86%), tetracycline (89.44-100%), erythromycin (83-88.89%), oxytetracycline (78.89%), sulfamethoxazole (100%), and ertapenem (66.67%), streptomycin (47.44-100%) and gentamicin (37.18%) *tetA* and *SHV* resistance genes were prevalent among the AMR *E. coli* isolates	[17,18,45,47-51]
*Salmonella* spp.	Cow dung, milk, milkers’ hand wash, soil, water, and vegetables of dairy farms; wild Irrawaddy squirrels	Mymensingh, Cox’s bazar and Bandarban	Amoxicillin (42.6%), erythromycin (87.5%), tetracycline (28.6-86.76%), oxytetracycline (75.73%) and ertapenem (50%), colistin sulfate (28.6%), cephalexin (71.4%) *tetA* and *SHV* resistance genes were prevalent among the AMR *Salmonella* isolates	[17,51]
*Bacillus* spp.	Milk from mastitis affected cows	Dhaka, Chattogram, Gazipur, Mymensingh, Sylhet, Satkhira, Rajshahi	Doxycycline, ampicillin, nalidixic acid, and erythromycin (60.0–84.0%), streptomycin (70-100%)	[18,45,47,48]
*Streptococcus* spp.	Milk from mastitis affected cows	Mymensingh, Rajshahi	Resistant to streptomycin (70-100%), amoxicillin (30-100%), and ampicillin (60-100%)	[45]
*Staphylococcus* spp.	Milk from mastitis affected cows; wild Irrawaddy squirrels	Dhaka, Chattogram, Gazipur, Mymensingh Satkhira, Rajshahi, Cox’s bazar and Bandarban	Amoxicillin (42-100%), ampicillin (73–100%), streptomycin (70-100%), tetracycline (30.8-88%), doxycycline (73.0–88.0%), ciprofloxacin (49.6%), colistin sulfate (53.9%), sulfamethoxazole-trimethoprim (30.8%), cephalexin (61.5%); chloramphenicol, ciprofloxacin, and nitrofurantoin (50.0–58.0%)	[18,45-48,51]
*Yersinia* spp.	Wild Irrawaddy squirrels	Cox’s bazar and Bandarban	Amoxicillin (69.2%), tetracycline (46.2%), colistin sulfate (53.9%), sulfamethoxazole-trimethoprim (30.8%), cephalexin (53.8%), ciprofloxacin (7.7%), gentamycin (15.4%)	[51]
*Klebsiella, Enterobacter, and Shigella*	Milk from mastitis affected cows	Dhaka, Mymensingh, Chattogram, Gazipur, Sylhet	Doxycycline, nalidixic acid, tetracycline, and ampicillin (70.0–100.0%); ciprofloxacin, gentamicin, nitrofurantoin, and chloramphenicol (30.0–70.0%).	[18]
*Pseudomonas aeruginosa*	Pus from abscess of cattle	Mymensingh	Resistant to ampicillin, oxytetracycline, tetracycline and amoxicillin	[52]
*Vibrio Cholerae*	Milk from cows, water and feces from farm environment	Mymensingh	Resistant to erythromycin (95.23%), azithromycin (76.9%) and ampicillin (52.38%)	[53]
Bacterial species not identified	Feces and waste-water of dairy farm and veterinary clinics	Dhaka	Bacterial isolates were significantly resistant to three aminoglycoside antibiotics-gentamycin, kanamycin and streptomycin	[19]
Bacterial species not identified	Bovine livers sold at retail meat shops	Chattogram	Tetracycline (100%), nalidixic acid (100%), oxacillin (100%), erythromycin (53.33%), ciprofloxacin (40%), doxycyclin (26.66%) and ampicillin (6.67%)	[54]

E. coli=Escherichia coli

MDR foodborne zoonotic pathogen *Salmonella* spp. carrying *tetA* and *SHV* resistance genes were reported from cow dung, milk, milkers’ hand, and dairy farm environment with resistance against a number of antibiotics such as erythromycin (87.5%), tetracycline (86.76%), oxytetracycline (75.73%), and ertapenem (50%) [17]. Dissemination of antibiotic-resistant *Salmonella* spp. even in wild Irrawaddy squirrels was identified. The wild Irrawaddy squirrels also harbored ABR *E. coli*, *Staphylococcus*, and *Yersinia* spp. All the antibiotic-resistant bacteria isolated from wild Irrawaddy squirrels showed resistance against amoxicillin, tetracycline, colistin sulfate, sulfamethoxazole-trimethoprim, and cephalexin [51].

Contamination of the animal originated food products by antibiotic-resistant bacteria with resistance against tetracycline (100%), nalidixic acid (100%), oxacillin (100%), erythromycin (53.33%), ciprofloxacin (40%), doxycycline (26.66%), and ampicillin (6.67%) was also reported [54].

### Institutional and policy structures to combat AMR in the veterinary sector

The state veterinary service of Bangladesh is provided through the DLS, Government of Bangladesh. DLS is providing veterinary service to the field level through its eight divisional, 64 district and 488 sub-district (Upazila) livestock offices. The veterinary hospital network of the country includes one central veterinary hospital, 64 district and 488 sub-district (Upazila) veterinary hospitals. In addition, there are nine metro veterinary hospitals to provide services in city areas. One to two registered veterinarians along with other supportive sub-technical personnel are deployed in each district, sub-district (Upazila) or metro veterinary hospitals [55]. The registered veterinarians provide animal healthcare services as well as engaged in monitoring and surveillance activities to ensure the prudent use of antimicrobials in the veterinary sector. In addition to veterinary hospitals, DLS has a sufficient number of laboratories for providing animal disease diagnostic supports to the field veterinarians and farmers. The laboratories are: One central disease investigation laboratory, eight field disease investigation laboratories, one quality control laboratory, and one veterinary public health and microbiology laboratory [55].

The Bangladesh government enacted “Fish Feed and Animal Feed Act, 2010” and “Animal Feed Rule, 2013” which prohibited the use of antibiotics in animal feed and restricted the use of colistin as a critically important antibiotic. These regulations empowered DLS to designate competent personnel for enforcing activities such as investigation, arrest, search, and seizure, prosecutions to prevent use of antibiotics in animal feed. Bangladesh is implementing the NAP for containing AMR in human, animal, and environment sectors through “One Health” approach where a coordinated effort from all implementing partners with strong commitment and participation are necessary for combating AMR in Bangladesh as well as to ensure one health. The NAP has assigned DLS to execute all necessary activities to achieve strategic objectives for containing AMR problems in veterinary practices. Although such activities are being performed by the DLS, there are still some policy gaps, including an explicit financing modality, specifications for antimicrobial stewardship in the veterinary sector, and rigorous operational, monitoring, and evaluation frameworks [6].

## Discussion

The animal health system of Bangladesh is explicitly “pluralistic” involving unqualified healthcare providers and different influential groups at different stages [13,23]. The absence of adequate, specifically state veterinary healthcare, regulatory and monitoring services, and low financial capability of the farmers has created pavements for the unlicensed village doctors, poultry and feed dealers, and pharmaceutical sales representatives to influence over the animal farm owners to take their decisions. In addition, increased demand for animal-source protein and knowledge gap on animal diseases, drugs, and AMR burden have driven the farmers toward the misuse or overuse of antibiotics. The weak regulatory regimes for antimicrobial stewardship programs and multidimensional anomalies in the use antibiotics in veterinary practices have resulted in the emergence of AMR pathogens in animals of Bangladesh [20]. Similar risk factors behind the emergence of AMR burden were previously reported in Southeast Asian and other LMIC [3,9].

The aforesaid risk factors have resulted in the emergence of distinguished AMR bacteria in different animal farming settings of Bangladesh (Tables-1 and 2). The circulations of AMR pathogens from January 2011 to June 2020 were reported by small-scale studies indeed carried out in different universities and covered only 13 districts out of 64 in the country. Therefore, the AMR situation in veterinary practices in most areas of the country remained unveiled. Even though, these small-scale studies reported a good number of AMR pathogens circulating in animals of Bangladesh with antibiogram profiling for each. For antibiogram profiling, most of the studies used culture and disk diffusion technique, although several studies incorporated molecular AMR genotyping tools [15-17,31]. Two studies adopted cutting edge technology – whole metagenome sequencing (WMS) along with disk diffusion technique to characterize the resistome of the microbiome of the clinical sample [18,42]. WMS approach coupled with bioinformatics is increasingly replacing conventional culture-dependent systems because of its capability to assessing the clonal diversity and similarity among human and animal bacterial isolates and providing greater insights into the shared resistance genes [4].

Insignificant difference (p=0.157, Pearson Chi-square test) between poultry and dairy farming settings with respect to AMR pathogen emergence deciphers that both types of farms are equally contributing to the emergence of AMR burden in Bangladesh. Of the AMR pathogens, MDR *E. coli* was the most abundant and *Salmonella* was the second most (Tables-1 and 2). The underlying causes may be their ubiquitous distribution and inclusion of *E. coli* as an indicator organism in most of the AMR studies [56]. High prevalence of MDR *E. coli* was also reported from poultry farms of Vietnam, India, and Ecuador [4]. Other than the organized poultry and dairy farming practices, the emergence of MDR *E. coli*, *Salmonella*, *Staphylococcus*, *Pasteurella*, and *Bacillus* spp. in unconventional poultry farming systems or MDR *E. coli*, *Salmonella*, and *Yersinia* species in small wild fauna are indicative of the high magnitude of the AMR burden in Bangladesh (Tables-1 and 2).

Among the MDR pathogens*, E. coli*, *Salmonella*, *Enterobacter*, *Staphylococcus*, and *Campylobacter* spp. are potential zoonotic pathogens creating direct human health hazards [57]. Unhygienic animal husbandry practices such as sharing of houses and water bodies with animals, disposal of farm wastes directly in the environment, along with poor personal hygiene of farm workers, and animal dwellers are exacerbating the risks of AMR dissemination in humans [13,58]. Moreover, contamination of broiler meat, frozen chicken meat, bovine meat, and milk, as well as the animal, originated food products with MDR bacteria, bringing the consumers closer to AMR hazards [17,18,44,54].

The reported MDR pathogens developed resistance against number of antibiotics commonly recommended to use in animal health [13,24] (Tables-1 and 2). The animal health is being directly affected by narrow-downed treatment scope resulted from high magnitude of AMR problem. Surprisingly, MDR *E. coli*, *Salmonella*, and *Enterobacter* isolated from both poultry and dairy also showed resistance against imipenem, azithromycin, and colistin sulfate recommended for human use only and strictly prohibited to use in animal health [17,22,41]. This may be due to use of these antibiotics in animal health or transmission of AMR genes from human to animal pathogens. The unethical use of antibiotics reserved for humans in veterinary practices will pose public health to a potential risk. The emergence of ESBL-producing genes and colistin-resistant *mcr-1* gene in ARB is alarming considering their potentiality to reduce the efficacy of last-resort antibiotics [15,34].

Although the DLS, Bangladesh has sufficiently extended its institutional establishments (veterinary hospitals and laboratories) throughout the country, these institutions are deficient in manpower, disease diagnostic facilities, and logistics. The veterinarians deployed in each of these hospitals are vested in providing service to a large jurisdiction which is practically impossible. Less availability of the state veterinary service often becomes a cause of “dissatisfaction” among the farmers [13]. Moreover, inadequate numbers of competent veterinarians at field level squeeze the scope of proper monitoring, surveillance, and AMR stewardship activities on AMU to containing this burden. The scarcity of proper animal disease diagnostic facilities in veterinary hospitals and laboratories is resulting in presumptive and wrong antimicrobial treatments; ultimately in AMR development. Despite some gaps, the policy structure of DLS is quite sufficient for combating AMR in veterinary practices of Bangladesh [6].

There is no alternative to formulating and executing an extensive and pragmatic surveillance program to unveil the real picture of the AMR situation in veterinary practices of Bangladesh. Scientific research to improve understanding of the risk factors for the emergence and spread of AMR is required to design evidence-based appropriate interventions. This review study revealed the AMR situation of a small area of the country and the inclusion of statistically significant number of districts in the surveillance program is recommended. Moreover, the surveillance program should include a survey on the type and amount of the antimicrobials used in different animal farming practices in Bangladesh. To explicit the emergence and distribution of the AMR pathogens in veterinary practices, laboratory-based AMR assessment in significant sample size from different farming settings is crucial. Research should address veterinary and human drug use, social, and economic influences on prescribing and drug-dispensing practices, traditional beliefs and local cultures, and environmental factors that promote the development of drug-resistant pathogens. The drug administration of Bangladesh should consider categorization of antimicrobials and preserve some items as prescription-only medicines. This will avoid availability or misuse of antimicrobial agents because pre-authorization or their use under supervision of infection experts will be required.

For effective monitoring, surveillance, AMR stewardship activities and awareness building on AMU and AMR, qualified manpower along with adequate funding is a prerequisite. Capacity building of veterinary hospitals and laboratories of the DLS with adequate manpower, training, logistics, and fund mobilization are crucial for successful containment of AMR in the veterinary sector of Bangladesh. However, we conclusively recommend the following measures for effective mitigation of AMR problems in veterinary practices of Bangladesh – (1) cross-sectorial policy commitment and coordination for the implementation of NAP; (2) development of the annual action plan, including national surveillance of AMR, awareness campaigns, and assessment of knowledge about AMR; (3) increasing regulatory role or law enforcement capacities of the implementing organizations; and (4) research on understanding the risk factors for the emergence and spread of AMR.

## Conclusion

AMR is considered one of the most important global health issues and Bangladesh is assessed as having a high risk of AMR. The emergence and zoonotic spread of ABR bacteria or associated-resistant genes is crucial because of their existence in animal farming environment, including soil, and water, animal products and by-products. AMR is a “One Health” problem, and its rational containment warrants inclusion of the veterinary sector. Thus, the present review study was envisaged to explicit the current situation, and knowledge gaps of AMR burden in the veterinary sector of Bangladesh. In this comprehensive review of the AMR situation in veterinary practices of Bangladesh, we tried to retrieve maximum available information. Nevertheless, due to limited open data sharing policy by some of the journals and restricted access to some of the databases, the search may not be exhaustive. Therefore, this review is limited by the inclusion of a small number of articles, even though sufficiently elucidated the underlying causes of AMR emergence, current situation of AMR in veterinary practices of Bangladesh along with the weaknesses and strength of DLS to contain the problem. Hence, this review article could be used as a reference work for formulation, adoption, and implementation of a rational and pragmatic future AMR containment program in veterinary practices in Bangladesh.

## Authors’ Contributions

MAA: Conceptualized and designed the review. MAA and MNH: Collected the literature. MAA: Analyzed the data. MAA and MNH: Drafted the manuscript. AZS, SS, and MMK: Edited and finalized the manuscript. MMK: Acquisition of funds. All the authors read and approved the manuscript.
